# Identification of Immunity Related Genes to Study the *Physalis peruviana* – *Fusarium oxysporum* Pathosystem

**DOI:** 10.1371/journal.pone.0068500

**Published:** 2013-07-03

**Authors:** Felix E. Enciso-Rodríguez, Carolina González, Edwin A. Rodríguez, Camilo E. López, David Landsman, Luz Stella Barrero, Leonardo Mariño-Ramírez

**Affiliations:** 1 Plant Molecular Genetics Laboratory, Center for Biotechnology and Bioindustry (CBB), Colombian Corporation for Agricultural Research (CORPOICA), Bogotá, Colombia; 2 Molecular Microbiology Laboratory, Center for Biotechnology and Bioindustry (CBB), Colombian Corporation for Agricultural Research (CORPOICA), Bogotá, Colombia; 3 Laboratorio de Fitopatología Molecular, Departamento de Biología, Universidad Nacional de Colombia, Bogotá, Colombia; 4 Computational Biology Branch, National Center for Biotechnology Information, National Library of Medicine, National Institutes of Health, Bethesda, Maryland, USA; 5 PanAmerican Bioinformatics Institute, Santa Marta, Magdalena, Colombia; Georgia Institute of Technology, United States of America

## Abstract

The Cape gooseberry ( *Physalis* *peruviana* L) is an Andean exotic fruit with high nutritional value and appealing medicinal properties. However, its cultivation faces important phytosanitary problems mainly due to pathogens like *Fusarium oxysporum,* *Cercosporaphysalidis* and *Alternaria* spp. Here we used the Cape gooseberry foliar transcriptome to search for proteins that encode conserved domains related to plant immunity including: NBS (Nucleotide Binding Site), CC (Coiled-Coil), TIR (Toll/Interleukin-1 Receptor). We identified 74 immunity related gene candidates in *P* *. peruviana* which have the typical resistance gene (R-gene) architecture, 17 Receptor like kinase (RLKs) candidates related to PAMP-Triggered Immunity (PTI), eight (TIR-NBS-LRR, or TNL) and nine (CC–NBS-LRR, or CNL) candidates related to Effector-Triggered Immunity (ETI) genes among others. These candidate genes were categorized by molecular function (98%), biological process (85%) and cellular component (79%) using gene ontology. Some of the most interesting predicted roles were those associated with binding and transferase activity. We designed 94 primers pairs from the 74 immunity-related genes (IRGs) to amplify the corresponding genomic regions on six genotypes that included resistant and susceptible materials. From these, we selected 17 single band amplicons and sequenced them in 14 *F. oxysporum* resistant and susceptible genotypes. Sequence polymorphisms were analyzed through preliminary candidate gene association, which allowed the detection of one SNP at the PpIRG-63 marker revealing a nonsynonymous mutation in the predicted LRR domain suggesting functional roles for resistance.

## Introduction

The Cape gooseberry ( *Physalis* *peruviana* L) is an exotic fruit from the Solanaceae family native to the Andean region which has spread to other parts of the world including Africa and India [1,2]. In addition to its high contents of vitamin A, C, B-complex [3] and minerals like iron and phosphorous [4,5], this tropical fruit is also known for its antioxidant [6], anticancer [3,7], anti-inflammatory [8,9], as well as diabetes and hypertension control properties [10]. Therefore the Cape gooseberry provides an enormous potential for biomedical research and commercial purposes [11]. Colombia is the first Cape gooseberry world producer followed by Zimbabwe, Malaysia, China, Kenya and South Africa [12]. However, Cape gooseberry production in the Andean region faces important phytosanitary problems due mainly to fungal diseases causing great crop losses and consequently a significant reduction in yield and quality. In Colombia, the most important fungal disease is the vascular wilt caused by *Fusarium oxysporum*, which may generate total crop losses [13]. Although the molecular defense mechanisms underlying plant–pathogen interactions have been studied extensively in a variety of pathosystems [14], little is known about the mechanisms underlying the *P* *. peruviana* – *F. oxysporum* interaction.

The current molecular model for plant immunity indicates the presence of two lines of defense: in the first one, plants sense conserved microbial molecular signatures called Pathogen-Associated Molecular Patterns (PAMPs) or Microbial-Associated Molecular Patterns (MAMPs) [15]. The recognition of these molecules is mediated by Pattern Recognition Receptors (PRRs), which are cell surface-located transmembrane receptors [15,16]. PRRs consist of extracellular Leucine-Rich Repeats (LRR) or Lysine Motif (LysM) domains. In some cases an intracellular Ser/Thr kinase domain is present [17,18,19]. This first level of recognition is known as PAMP-Triggered Immunity or PTI [20] and it can confer broad-spectrum resistance and durable resistance against different types of non-host or non adapted pathogens [21]. However, successful pathogens evade or suppress the PTI through the translocation of effector proteins into the plant cell [16]. In turn, plants have evolved to directly or indirectly recognize such effectors through additional receptors called resistance (R) proteins [22]. This second layer of defense is known as Effector-Triggered Immunity (ETI), and is often accompanied by Hypersensitive Response (HR), blocking pathogen spread [23]. The majority of R proteins have a modular structure characterized by a conserved central Nucleotide Binding Site (NBS) and a more variable C terminal LRR domain [24,25]. The amino-terminal region present in NBS-LRR proteins has allowed their classification into two major classes defined by the presence of Toll/Interleukin-1 Receptor (TIR) or Coiled Coil (CC) domains [25,26,27]. Sets of candidate disease resistance genes have been identified in model species with complete genomes using the domains described above. Accordingly, 149 NBS-LRRs genes have been identified in *Arabidopsis* , 875 in Rice and 400 in *Populus* [28,29,30]. Additionally, the presence of the NBS domain in non-model species has become critical for the association of genomic sequences with disease resistance function [31,32,33,34,35].

Expressed Sequenced Tags (ESTs) analyses are critical for the discovery of novel genes like those involved in PTI and ETI, particularly in non-model plants for which a complete genome is currently not available as in the case of the Cape gooseberry. We recently characterized the Cape gooseberry foliar transcriptome and have developed microsatellite markers for plant improvement in this species [36,37]. This manuscript provides the first description of a computational strategy to identify putative immunity related genes (IRGs) encoded in the *P* *. peruviana* foliar transcriptome used to design primers to PCR amplify a number of IRG fragments in a pool of *F. oxysporum* resistant and susceptible genotypes. We were able to identify a set of polymorphisms (SNPs) initially related to resistance, this finding is the basis for further experimentation that will include larger populations and markers to contribute future marker assisted selection in Cape gooseberry.

## Materials and Methods

### 1: Plant material and DNA isolation

Cape gooseberry and related taxa genotypes (Table 1) were selected from the Colombian Corporation for Agricultural Research (CORPOICA) germ plasm collection, based on resistance and susceptibility responses against *F. oxysporum* (Table S1). Plants were propagated *in vitro* and maintained in growing chambers at 20°C. Genomic DNA was isolated from young leaves, following a previously described methodology [38] with some modifications. Briefly, approximately 500 mg young leaves were ground using a mortar and pestle with liquid nitrogen and incubated at 65^°^C for 30 min in extraction buffer (0.15M Tris-HCl pH 8, 0.01M EDTA pH 8, 1% CTAB, 1% PVP 360000 and 5% β-mercaptoethanol). Samples were centrifuged at 18,000 g and the supernatant was extracted twice with one volume of phenol-chloroform-isoamyl alcohol (25:24:1). Next, the supernatant was precipitated with isopropyl alcohol and washed with 70% ethanol. Pellets were dissolved in 1X TE buffer, treated with RNase A (10 mg/mL) at 37^°^C for 20 min and stored at -20°C until use. The quantity and quality of DNA was checked using a NanoDrop® ND-1000 Spectrophotometer and on 1% (w/v) agarose gels stained with ethidium bromide (0.5 µg/mL).

**Table 1:** Genotypes of Cape gooseberry and related taxa used in this study.

**Species**	**Accession – Genotype Number**	**Repository - Country of origin**	Response to F. oxysporum^**^	**State of cultivation**
*Physalis* *peruviana*	09U047-1^β ε^	Corpoica^δ^-Colombia	5,0	Weedy
	09U047-4^§ε^			
*Physalis* *philadelphica*	09U063-7^ε^	Corpoica-Guatemala	9,0	Wild
*Physalis* *philadelphica*	09U071-4^ε^	Corpoica-Guatemala	9,0	Wild
*Physalis* *peruviana*	09U086-4^ε^	Corpoica-Ecuador	5,7	Weedy
*Physalis* *peruviana*	09U089-1^§ βε^	Corpoica-Colombia	5	Elite^†^
*Physalis* *peruviana*	09U099-1^§ε^	Corpoica-Colombia	4,7	Elite
*Physalis* *floridana*	09U139-1^ε^	Birmingham Botanical garden	9,0	Wild
*Physalis* *floridana*	09U141-1^§ε^	Corpoica	9,0	Wild
*Physalis* *angulata*	09U173-3^ε^	Corpoica-Colombia	9,0	Wild
*Solanum* *auriculatum*	09U178-4^§ε^	Corpoica-Ecuador	0	Wild
*Physalis* *peruviana*	09U210-6^ε^	Corpoica	5,0	Cultivated
*Physalis* *peruviana*	09U216-6 ^ε *^	Corpoica	5,8	Cultivated
*Physalis* *peruviana*	09U274-3^ε^	Corpoica	5,7	Elite
*Physalis* *peruviana*	09U279-4^§ε^	Corpoica-Colombia	2,0	Weedy

^β^ Genotypes used for Sanger sequencing;^ε^ Genotypes used for 454 sequencing; ^§^ Genotypes used to confirm success of primer design; ^*^ Genotype used for foliar transcriptome sequence [37]; ^δ^ Colombian Corporation for Agricultural Research;^**^ See scale on Table S1;^†^ Commercial material used for export markets or Landrace material cultivated by farmers.

### 2: Evaluation of the resistance phenotype

Fifteen genotypes from *P* *. peruviana* and related taxa (Table 1) were challenged with a *F. oxysporum* pathogenic strain (Map 5) isolated from *P* *. peruviana* and supplied by the *Fusarium* Collection at Corpoica’s Molecular Microbiology Laboratory. For inoculum production, the monosporic strain Map 5 was reactivated in Potato Dextrose Agar (PDA) medium (BD Franklin Lakes, NJ) for 15 days at 28°C. Then, it was grown in liquid Potato Dextrose Broth (PDB) (BD Franklin Lakes, NJ) for ten days at 28°C in constant agitation (140 rpm); the inoculum was prepared according to Namiki et al. [39] and adjusted to desired final concentration. Once *P* *. peruviana* seedlings had a pair of true leaves and were 5 to 7 cm tall, 12 seedlings of each genotype were transplanted individually into plastic pots with 255 g sterilized soil-rice husk substrate 3:1 ratio and were then sowed in a completely randomized design under field conditions in the year 2011 in Mosquera, *Cundinamarca* , Colombia located at 2,516 meters above sea level. Plants were inoculated with a conidial suspension using one as a control in sterile water. The inoculation was done by the root dip method [39]. Briefly, the roots were dipped in 75 mL of spore suspension (1x10^5^ CFU/mL) for three minutes and were transplanted into the same pots. External symptoms were scored 2 weeks after inoculation during 45 days. The severity degree of the disease was registered daily using a scale of symptoms proposed for the pathosystem *Physalis* *peruviana* -*Fusarium oxysporum* (Supplementary Table S1). The scale of symptoms was based in other scales [39,40,41,42].

### 3: Computational identification of candidate resistance genes (Immunity Related Genes-IRGs)

A complete set of plant protein sequences was obtained from the NCBI Protein (http://www.ncbi.nlm.nih.gov/protein) and the SOL Genomics Network (SGN) [43] databases. All proteins retrieved were clustered using Blastclust (ftp://ftp.ncbi.nih.gov/blast/) to eliminate redundancy. We used the Pfam database [44] to retrieve a HMM profiles from NB-ARC (PF00931), TIR (PF01582), LysM (PF01476) and the Pkinase domain (PF00069). These profiles were used to search all plant proteins using HMMER version 3.0 [45], and the predicted protein architecture was validated using RPS-BLAST (E-value cutoff <1e-4) and the Conserved Domain Database. This procedure generated a collection of plant resistance protein database. The CC domain identification was performed by a standard method [46]. Additionally, since the LRR domain is highly variable LRRfinder [47] was selected as the method of choice for LRR identification. The collection of the Cape gooseberry leaf transcript sequences (Transcriptome Shotgun Assembly (TSA) Database, GenBank Accession numbers JO124085-JO157957) was used as the source for plant resistance transcript identification in this species using TBLASTN and the plant resistance protein database described above.

### 4: Annotation of candidate resistance genes in Cape gooseberry

The Cape gooseberry resistance and immunity related transcripts identified above were compared with the UniProtKB/Swiss-Prot database [48], using BLASTX [49]. The top five protein hits for each query were submitted for functional classification using Blast2GO [50]. GO terms were assigned employing three Gene Ontology categories: cellular component, molecular function and biological process [51].

### 5: Primer design and PCR amplification

We used the *Solanum* *lycopersicum* genome and the Cape gooseberry mRNA to identify possible intron and exon regions using NCBI’s Spidey (http://www.ncbi.nlm.nih.gov/spidey) for primer design at intron-exon boundaries in all immunity related transcripts. Primers were designed using Primer3 [52] and subsequently checked for self-complementarity, hairpins and dimers. PCR amplification was carried out in an i-Cycler thermal Cycler (Bio-Rad, Hercules, CA, USA) as follows: one cycle of initial denaturation for 5 min at 94°C, followed by 35 cycles for 30 s at 94°C, 1 min at 54°C and 2 min at 72°C, followed by a final extension of 10 min at 72°C, and preserved at 4°C until further analysis. To verify PCR amplification success of the designed primers, we tested them on six Cape gooseberry and related taxa genotypes with variable resistance responses (Table 1). Amplification products were separated by gel electrophoresis on 2% (w/v) agarose in a 1X TAE buffer (40 mM Tris-acetate and 1 mM EDTA), and then stained with ethidium bromide (0.5 µg/mL).

### 6: PCR sequencing and analysis

Seventeen PCR products from IRGs were selected based on single band amplification on the six genotypes tested, and were sequenced initially by single pass, using forward and reverse primers for each amplicon in two of the six genotypes (09U47-1 and 09U89-1) using the same PCR conditions as described above with commercial Taq DNA polymerase (Invitrogen, San Diego, CA). Each read was processed with a variant call pipeline [53] that uses Phred/Phrap/Consed for base calling and assembly [54,55].

Then, we amplified the 17 IRGs on the set of 15 genotypes from 14 accessions (Table 1), and sequenced them using the 454 GS-FLX Titanium platform (Roche Diagnostics Corporation). The raw reads were deposited in the NCBI Sequence Read Archive (accession number SRX216233). Reads for all genotypes and markers were trimmed using Mothur [56], aligned with BWA [57], using as consensus the sequence produced by Sanger sequencing. SNP calling was achieved using Samtools [58] with default parameters. We performed a General Lineal Model (GLM) procedure using the Tassel version 3.0.146 [59] with a Minimum Allele Frequency (MAF) of 0.1 and removing minor SNP states in order to avoid false positives. The analyses took into account the structure and ancestry of the sample population by using Q matrix for each genotype generated by Structure 2.3.3 [60]. The K parameters ranged from *K* = 1 to *K* = 10 across ten runs with 10^6^ iterations and a burning period of 500,000. Bonferroni corrections were performed to establish *P value* cutoffs of α = 0.01 and α = 0.05.

## Results

### Generation of a plant immunity related protein database

**Figure 1:**
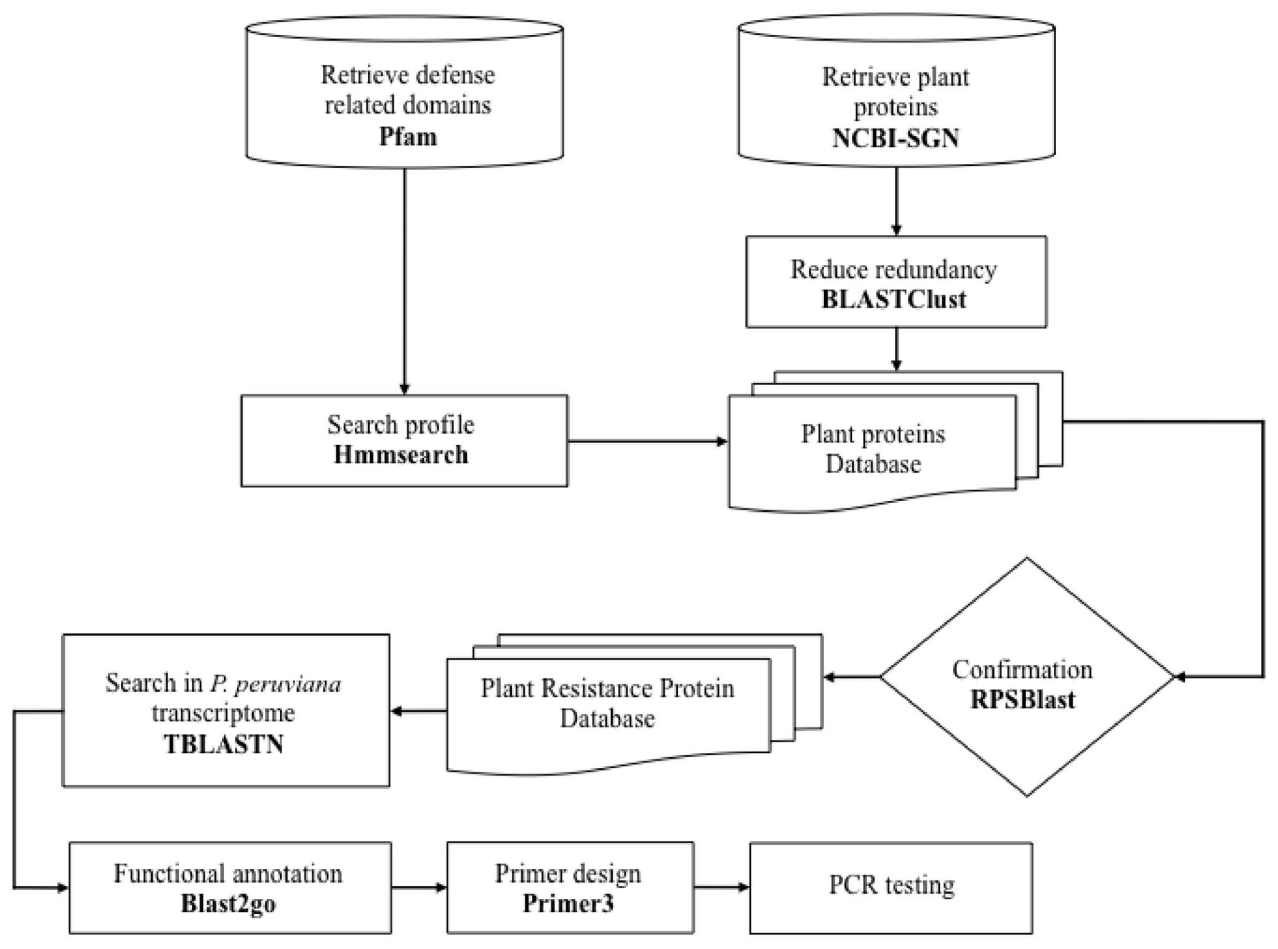
Pipeline used to search for resistance related domains in *Physalis* *peruviana* foliar transcripts.

**Figure 2:**
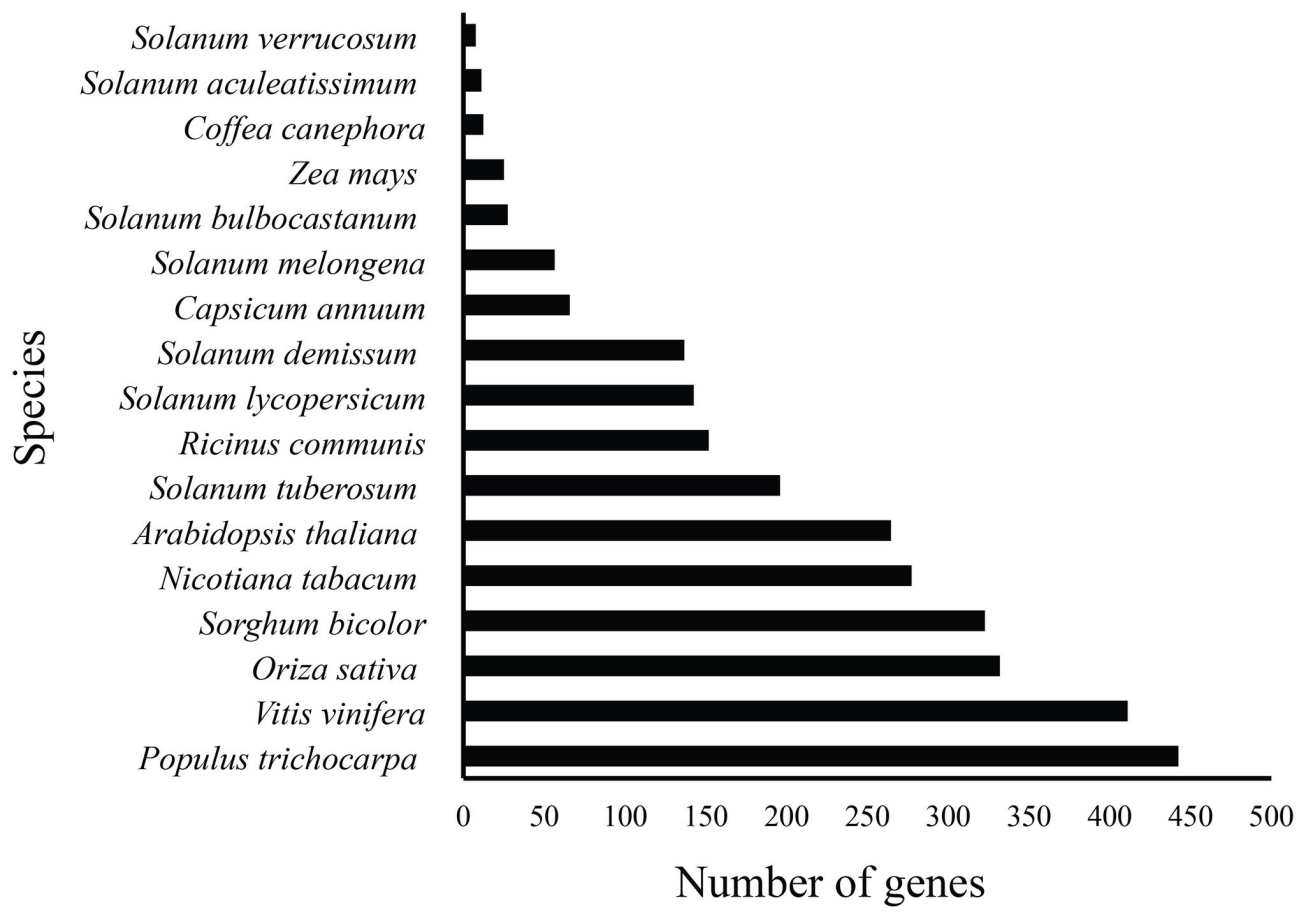
Number of candidate resistance genes related to plant immunity found in model and in non-model plant species.

We generated a curated plant immunity database with a total of 3,691 proteins using a methodology described in Figure 1. These proteins were identified mainly in model species like *Populus* *trichocarpa* , *Vitis vinifera* and *Oryza sativa* as well as other non-model species like *Solanum* *demissum* , *Solanum* *bulbocastanum* (Figure 2). Nearly all proteins contain NBS domains (3,456), which are associated with TIR (590), CC (959) or Pkinase (61) domains, and others were associated with LysM and LRR (62 and 1017 respectively, Table 2). Notice that one of the most abundant architectures presented here is CC–NBS-LRR, known to be associated with Effector-Triggered Immunity (ETI).

**Table 2:** Protein architecture of plant resistance genes identified in a plant protein database created from databases at NCBI and SGN.

**Protein Architecture**	**Number of proteins**
LRR	126
LRR-Pkinase	11
LysM	29
LysM-Pkinase	33
NBS	1596
NBS-Pkinase	6
TIR	33
TIR-NBS	115
NBS-LRR	392
NBS-LRR-Pkinase	3
TIR-NBS-LRR	384
TIR-CC-NBS	17
TIR-CC-NBS-LRR	40
TIR-NBS-LRR-Pkinase	1
CC–NBS-LRR	452
CC–NBS-Pkinase	4
CC–NBS	446
Pkinase	3
**Total**	**3,691**

### Identification of transcripts related to plant immunity in Cape gooseberry

Similarity TBLASTN searches using the plant immunity related protein database described here were carried out on the Cape gooseberry transcriptome with an e-value cutoff of 1e-4 [49], obtaining a total of 74 isotigs with hits (Table 3). We successfully identified immunity related transcripts similar to others reported in model species even though the tissue used was not inoculated with a pathogen. A total of 42 isotigs were associated with the NBS domain, 11 with TIR, 15 with CC, 48 with LRR, 21 with Pkinase and 5 with LysM. Therefore, we report 19 candidate transcripts encoding typical domains related with the first layer of defense (PTI). Among the PTI candidate transcripts, 17 of them (14 LPk and three LysMPk) were identified as RLKs (Receptor like kinase), and only two (LysM) as RLP (Receptor like protein), which have been reported as an important PRR to recognize fungal pathogens [61] like *F. oxysporum*. Regarding the second layer of immunity or ETI, we identified a total of 45 isotigs associated mainly with the NBS domain. Among these genes, 10 were identified as NL (NBS-LRR), eight as TNLs (TIR-NBS-LRR), and nine as CNLs (CC-NBS-LRR). In addition, 18 candidate transcripts that lack a canonical architecture typical of resistance genes were classified as N with 10 candidates, T with two, C with one, CN with four as well as the non-common architecture TCNL with one transcript (Table 3).

**Table 3:** Predicted domain architecture of resistance gene candidates in *P* *. peruviana* related to the first and second layer of defense.

**Predicted Protein Domains**	**Letter code**	**Isotigs with Hits**	**Defense layer**
LRR-Pkinase	LPk	14	PTI
LysM	LysM	2	
LysM-Pkinase	LysMPk	3	
NBS	N	10	ETI
NBS-LRR	NL	10	
TIR	T	2	
TIR-NBS-LRR	TNL	8	
TIR-CC-NBS-LRR	TCNL	1	
CC	C	1	
CC–NBS	CN	4	
CC–NBS-LRR	CNL	9	
Other*		10	
**Total**		**74**	

* Additional domain LRR (six isotigs) and Pkinase (four isotigs) that might be implicated in processes other than defense.

### Functional annotation of resistance related transcripts

**Figure 3:**
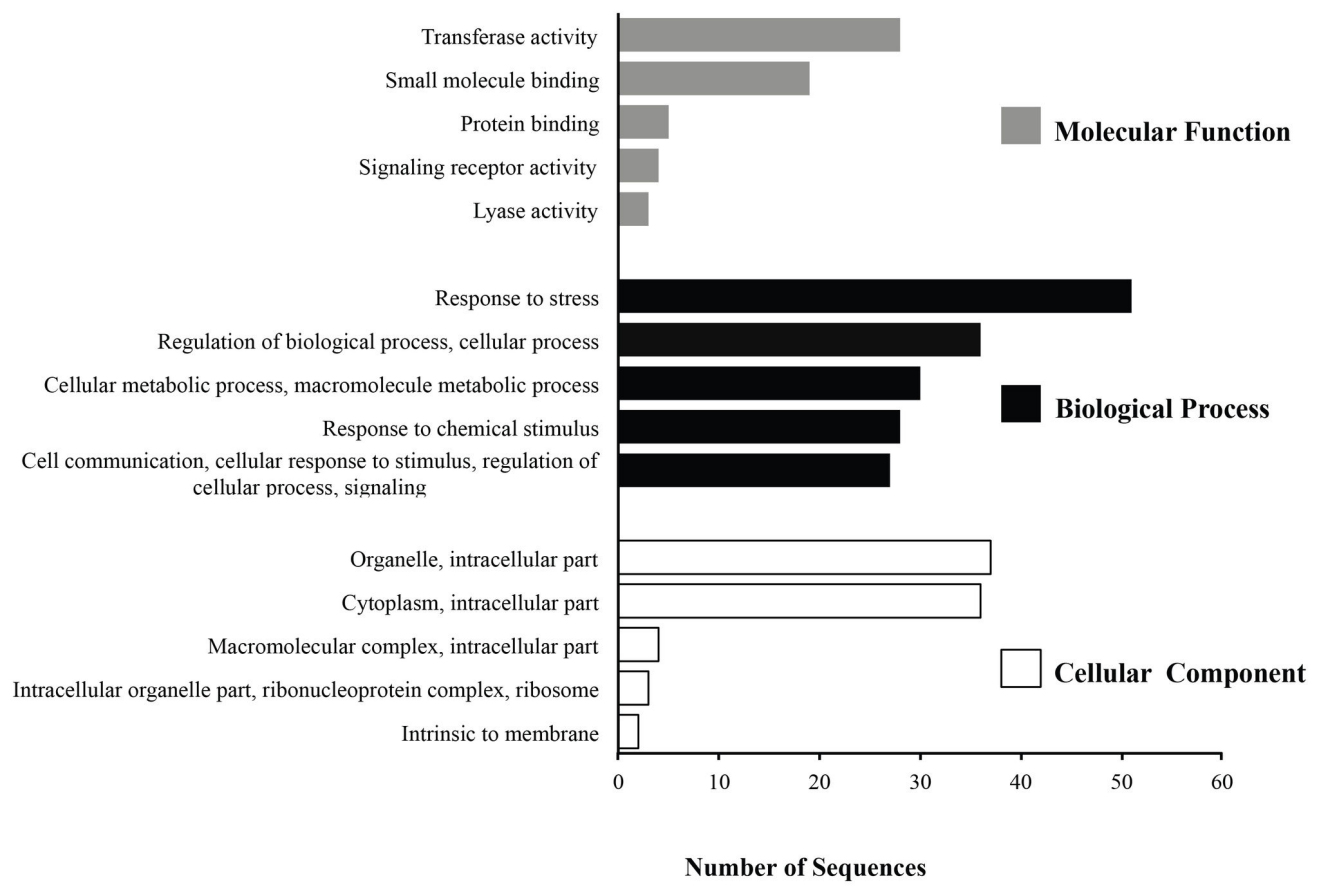
Functional distribution of resistance related isotigs based on gene ontologies: molecular function, biological processes and cellular component in the Cape gooseberry transcriptome. Only major hits are shown (E-value ≤ 1E-4) for each GO category.

We assigned functional gene ontology categories [51] to all 74 isotigs with sequence similarity to immunity related proteins based on the presence of conserved domains. Each isotig was compared to the UniProtKB/Swiss-Prot database and GO terms were assigned to each query using Blast2GO [50]. A total of 1047 ontologies were associated with 68 sequences annotated and assigned to the three principal categories. For Biological process, 85% of genes were associated mainly with response to stress and regulation of biological process. For Molecular function and Cellular component, 98% and 79% genes were associated mainly with transferase activity and small molecule binding for the first one, and cell organelle and cytoplasm for the second one (Figure 3). The remaining six sequences did not show similarity to known proteins and were not assigned to any GO categories.

### Identification of polymorphisms at IRGs and their relationship to *F. oxysporum* resistance

We designed 94 primer pairs from 74 isotigs with sequence similarity to immunity related proteins. In some cases we designed additional primer pairs per isotig in which more than one intron-exon boundaries were predicted. A total of 85 primer pairs (90%) produced amplification products on six genotypes (Supplementary Table S2).

From the 17 454-sequenced IRGs on 15 genotypes, we could process 14 of them, since the genotype 09U178-4 had more than 60% missing data probably due to the fact that it belongs to another genus ( *Solanum* ), therefore, it was eliminated from further analyses. Read coverage for each amplicon ranged from 0 to 401 reads per IRG (Supplementary Table S3). We obtained a multi-fasta alignment with an average 7804 nt per genotype and 109,256 nt over all sequences, identifying a total of 213 SNPs filtered with a MAF = 0.1.

**Figure 4:**
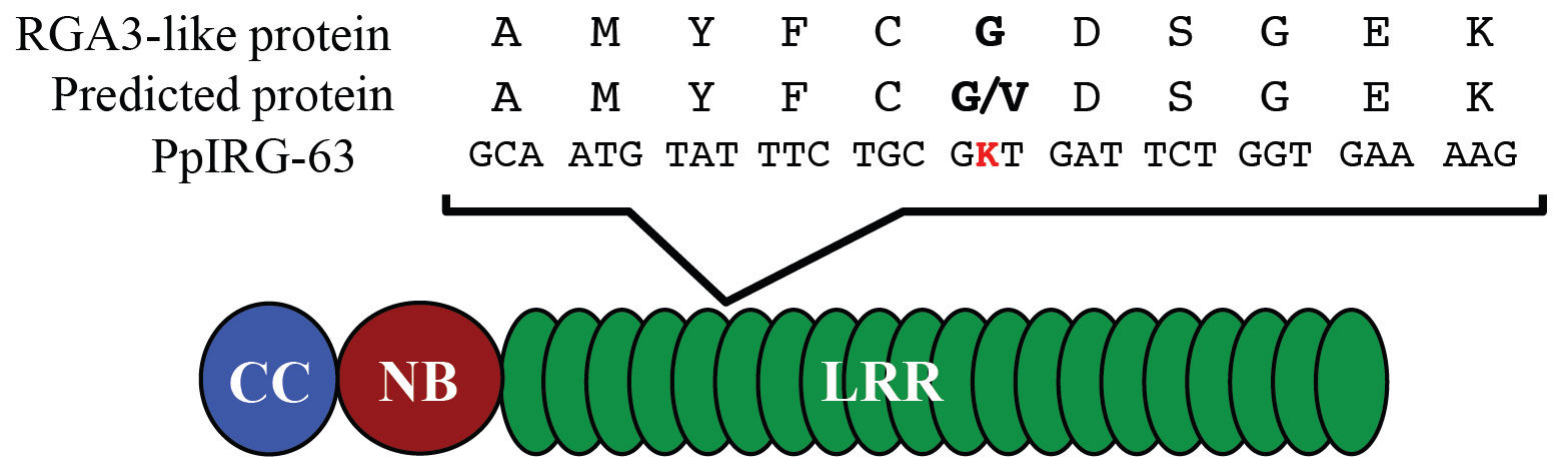
Single nucleotide polymorphisms (SNPs) detected in the PpIRG-63 marker located on the predicted LRR domain. Polymorphisms are shown in red.

To reduce spurious associations between the SNPs from IRGs and the resistance trait, we performed structure analyses on the tested population sample. The analyses revealed two subpopulations using the ΔK method [62]; the first was formed by related taxa ( *P* *. floridana* and *P* *. angulata* genotypes) and the second clustered all the *P* *. peruviana* genotypes. The clusters formed were not related to resistance against *F. oxysporum* nor to the place of origin or state of cultivation, they were only related at the species level. Next, preliminary candidate gene association analyses were performed using GLM from structure results as well as the SNP and resistance phenotype data. After Bonferroni correction, significant association was found in one SNP (SNP_483), with a marker *p value* = 1.72E-6 at α = 0.01 level (*p value* cutoff = 4.69E-5) and α = 0.05 level (*p value* cutoff = 2.34E-4). This SNP is located at the PpIRG-63 corresponding to a CC–NB-LRR predicted protein RGA3-like (Figure 4).

## Discussion

Cape gooseberry ( *P* *. peruviana* L.) is an important commercial crop with high nutritional value and interesting medicinal properties. This study constitutes an initial effort for future improvement of Cape gooseberry cultivars against pathogens like *F. oxysporum*. Molecular breeding to improve Cape gooseberry materials for resistance or tolerance against different diseases require the identification and isolation of immunity genes for incorporation into susceptible commercial cultivars using different strategies such as marker assisted selection, transgenesis or directed mutagenesis, which might advance towards genome wide selection [63], as more resistant phenotype and marker data becomes available for the species. These strategies are still under development for this orphan species, however existing transcriptome data available [37] is being used effectively to identify markers and immunity related genes for future development efforts.

Immunity related genes, which confer resistance to diverse pathogens like fungi, bacteria and viruses have conserved domains where the most common are LRR, LysM and kinase domains present in the PRR proteins involved in PTI. The R proteins are characterized by the presence of LRR and NBS domains that can be accompanied by a TIR or a CC domain located at their N-termini. The presence of conserved domains, particularly NBS have allowed development of strategies based on degenerate primers to amplify genes related to plant immunity in different species [31,32,34]. With the expansion of complete genome sequences or transcriptomes it is possible to identify genes coding for proteins with these conserved domains [64]. The number of these types of genes is variable in different species. In *Arabidopsis* for example there are 149 NBS-LRR genes, 875 in rice, 738 in potato, 400 in *Populus* , 630 in sunflower, 92 in *Brassica* among others [29,30,65,66,67,68].

**Figure 5:**
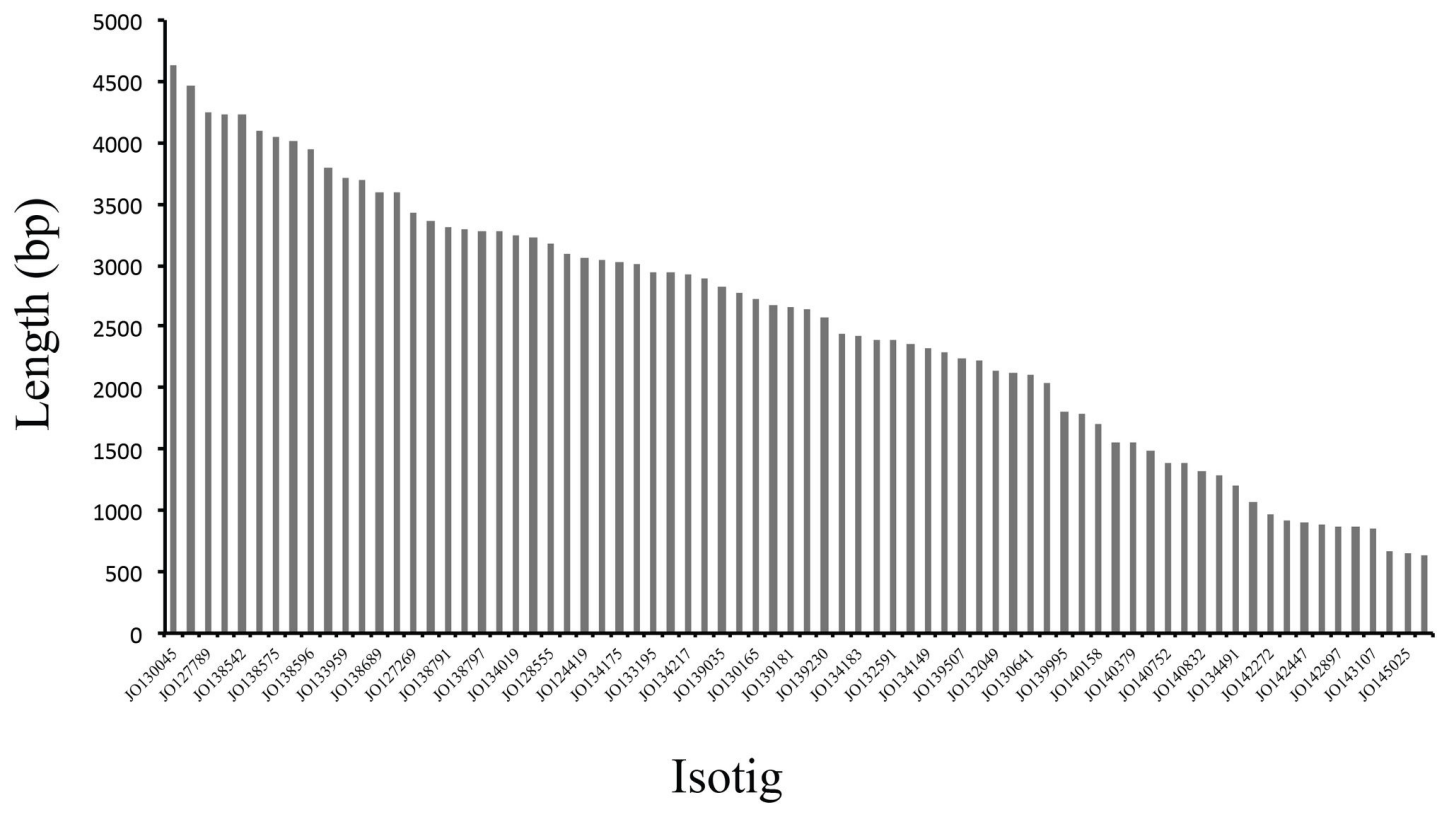
Isotig size distribution for immunity related genes in Cape gooseberry.

In this study we identified 74 transcripts related to plant immunity where the majority are represented by NBS and LRR domains (58.1% respectively). It is possible that some transcripts do not represent the full-length mRNA or the complete plant resistance gene architecture. This statement is raised because of the fact that most of the immunity related isotigs reported here had a size less than 4,640 bp (Figure 5). Thus, in most cases we were only able to identify incomplete proteins with classic TNLs or CNLs domain architectures. Nonetheless, these gene fragments were still useful for studying function *in silico* and for preliminarily identifying polymorphisms related to resistance.

Most immunity genes, notably those coding for NBS-LRR proteins, are constitutively expressed [69]. Although we used foliar tissue that is not usually infected by *F. oxysporum*, we successfully identified candidates with typical plant resistance gene architectures that are useful for initial screenings of different resistant and susceptible sources in germ plasm collections. Some of the genes related to plant immunity reported here, are widely recognized for their response against plant pathogens in model plants (Table 4). Genes like Prf, Tm2 and BS4 had the lowest TBLASTN E-values indicating high similarity to their Cape gooseberry counterparts.

**Table 4:** Resistance genes reported in model organisms with significant hits in the Cape gooseberry transcriptome.

**Protein ID** ^§^	**Domains**	**Isotig**	**Gene/Protein Name**	**Species name**	**BLAST E-value**
4689223	CC–NBS-LRR	JO142447	I2	*Solanum* *lycopersicum*	1E-102
8547237	CC–NBS	JO133481	Prf	*Solanum* *lycopersicum*	0
38489219	TIR-NBS	JO132049	BS4	*Solanum* *lycopersicum*	2E-142
558887	TIR-NBS-LRR	JO129083	N	*Nicotiana* *glutinosa*	8E-7
56406364	CC–NBS	JO138325	Tm-2 ToMV resistant protein	*Solanum* *lycopersicum*	2E-163

§ GenBank protein ID.

In tomato, the I2 gene confers full resistance against *F. oxysporumlycopersici* race 2 [70]. I2 is present as a monophyletic group in the Solanaceae family, leading to the conservation of binding and hydrolysis functions at the NBS domain, suggesting that its function is maintained in many members of the I2 family [71]. Besides, the relatively slow rate of birth-and-death in the I2 family and because of the fact that new I2 duplicates acquire novel functions or become specialized representing functional copies on distant phylogenetic species from potato to tobacco [71]; we might expect that the homologous I2 gene found in Cape gooseberry could be functional and possibly detect effectors secreted by *F. oxysporum* on *P* *. peruviana* . Similarly, we found homologs for Prf, BS4, and N identified in Solanaceae species (Prf and BS4 in *S* *. lycopersicum* and N in *Nicotiana* *glutinosa* , Table 4), therefore we might expect as with I2, their conservation in Cape gooseberry as functional PRR, R genes and defense proteins like the ones in tomato and other Solanaceae species since this family is characterized by a high conservation of gene content and order and affected by many of the same pathogens [72].

On the other hand, our results are consistent with the role and localization of the proteins involved in plant resistance, as evidenced by the functional annotation using Blas2GO. For example, the NBS domain, characteristic of the R proteins is involved in plant defense through direct or indirect recognition of virulence factors [24]. This domain is required for ATP and GTP binding [73], as reported for I2 and Mi-1 in tomato modulating the binding and hydrolysis of ATP in the signaling cascade of resistance [74]. Additionally, the NBS domain has been related to nucleotide phosphatase activity in a subset of R genes and seems that its biochemical activity co-evolved with the plant resistance signaling pathways [75]. On the other hand, RLKs are related to transmembrane signaling receptor activity, through PAMP/MAMPs recognition trigger defense responses [15].

Here we report one SNP mapped to the PpIRG-63 marker, preliminary associated with the resistance response against *F. oxysporum* (Figure 4). This gene is involved in the second layer of plant resistance and has a typical R gene architecture CC–NB-LRR (Supplementary Table S2), representing a potential plant immunity gene in Cape gooseberry. The SNP_438 is present at the exonic region in the predicted LRR domain, which is involved in protein–protein interactions and ligand binding [76]. This domain is believed to be the major determinant of effector recognition [77]. In several plant–pathogen systems, the sequence variation in the LRR domain, particularly in the β stand/β turn motif (xxLxLxx motif) has been shown to be responsible for different recognition or resistance specificities [78,79]. As demonstrated in rice, the knowledge of the variation patterns of R-genes through the study of allelic diversity in NBS-LRR genes, is of fundamental importance for plant breeders attempting to preserve resistant germplasm [80]. These observations bolster the possibility that the SNP polymorphism present in the PpIRG-63 related with plant immunity could be associated with pathogen recognition in *P* *. peruviana* .

Interestingly, the SNP described here is a non-synonymous substitution (valine to glycine) and both encode nonpolar amino acids. This kind of substitution is frequently present in NBS-LRR proteins maintained by diversifying selection [25] as a possible response to new variants of the pathogen. Whether this polymorphism is causal to resistance against *F. oxysporum* awaits further investigation through association studies with larger populations and markers or functional approaches.

## Conclusion

This study is the first report on the discovery of genes putatively related to plant immunity in Cape gooseberry. We reported 74 genes related to the first and second layer of plant pathogen recognition found in the *P* *. peruviana* foliar transcriptome. We identified genes with the typical R architecture and found 17 RLKs candidates related with PTI, eight TNLs and nine CNLs candidates related with ETI. Functional annotation using gene ontologies predict their roles in resistance against plant pathogens. Ninety-four primers were designed from these candidate genes, but no InDels (>50 bp) were found between resistant and susceptible plant genotypes. We sequenced, 17 IRGs to perform preliminary candidate gene association analyses on a set of 14 genotypes. One marker (PpIRG-63) revealed a non-synonymous SNP polymorphism (SNP_438) in the LRR predicted domain and was preliminary associated with resistance to *F. oxysporum*, representing the first pathogen resistance candidate gene known in this pathosystem. Further association analyses using larger population sizes would be needed to determine its function as molecular marker for breeding and phytosanitary programs in *P* *. peruviana* .

## Supporting Information

Table S1 Severity scale of symptoms for the *Physalis* *peruviana* *-* *Fusarium* *oxysyporum* pathosystem.
(DOCX)

Table S2 Primers designed for the identification of immunity related genes in Cape gooseberry.
(DOCX)

Table S3 Number of reads per marker sequenced by 454 on the 14 *Physalis* genotypes.
(DOCX)
