# Supplementary material for: Identification of Immunity Related Genes to Study the Physalis peruviana – Fusarium oxysporum Pathosystem
Source: PLoS One. 2013 Jul 3;8(7):e68500. doi: 10.1371/journal.pone.0068500 (PMC3701084; doi:10.1371/journal.pone.0068500)
Supplement: Table S1 — Severity scale of symptoms for the Physalis peruviana - Fusarium oxysyporum pathosystem. (DOCX) [file pone.0068500.s001.docx]

**Table S1**: Severity scale of symptoms for the *Physalis peruviana - Fusarium oxysyporum* pathosystem.

| **Category Name** | **Degree** | **Severity(%)** | **Main Symptoms** | **Phenotype** |
| --- | --- | --- | --- | --- |
| 0. Resistance | 0 | = 0 | Any visible symptoms | 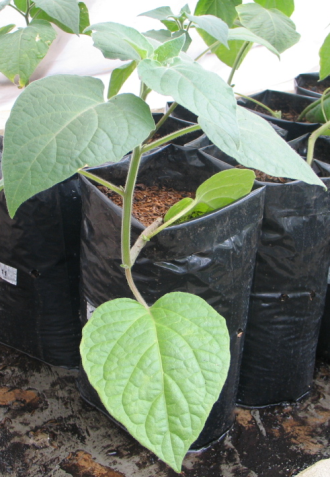 |
|  | 1 | ≤ 5 | Few leaf damage, low to moderate discoloration |  |
| 1. Low susceptible | 2 | ≥ 6 - ≤10 | Few lesions on the leaves; discoloration to light green with many dark green areas | 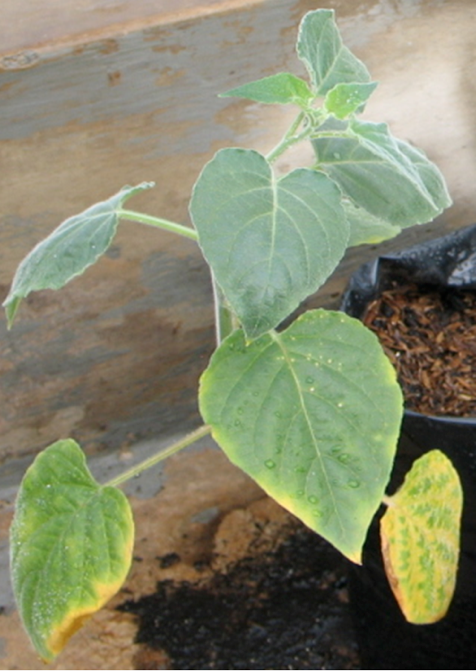 |
|  | 3 | ≥11-≤ 20 | Few lesions, wilting on the edge of the leaf; shortly infected leaves, pale green or slightly yellow, with loss of turgor. |  |
| 2. Moderately susceptible | 4 | ≥21-≤ 40 | Evident infection in the leaves, discoloration to pale yellow, dried leaf edges, brown "burned", with little or no turgor, moderate infection of the stem; chlorosis. | 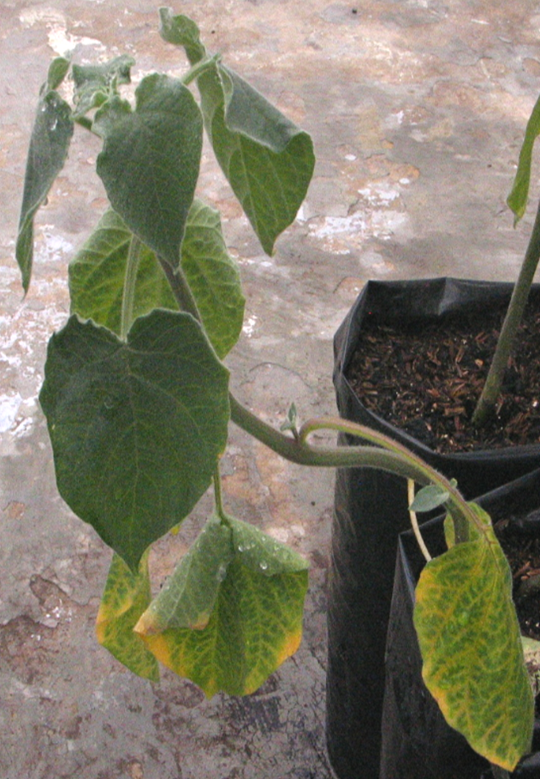 |
|  | 5 | ≥41-≤ 60 | Severe infection of leaves, yellow ocher shades, dried leaf to the midrib, "burned" edges; prostration of pedicel; total loss of turgor, moderate infection on the stem and purple color on the basis of the stem, chlorosis and / or necrosis. |  |
| 3. Susceptible | 6 | ≥61-≤ 70 | Severe lesions on the leaves, "Burn" overall, wilting, chlorosis, necrosis and / or premature defoliation; prostration of the stem, purple stain on the basis of the stem. | 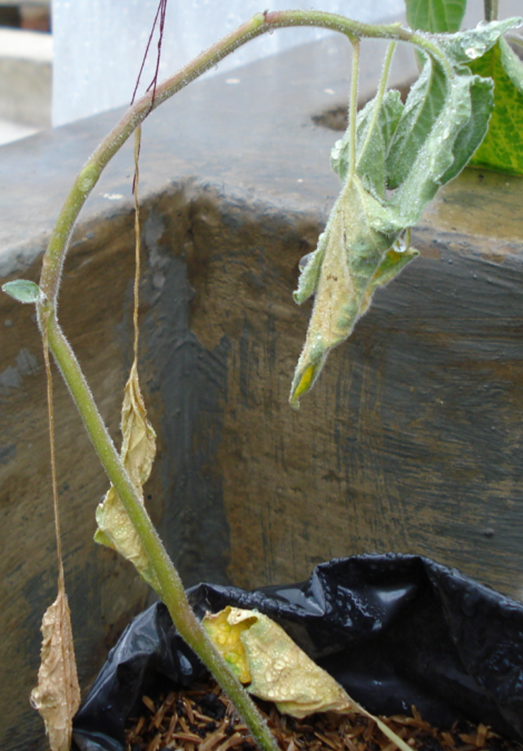 |
|  | 7 | ≥71-≤ 80 | Dead leaves or 100% wilting, chlorosis, necrosis and / or premature defoliation, severe prostration, purple or violet stain on the stem. |  |
| 4. Highly susceptible | 8 | ≥81 -≤ 90 | Dead leaves, wilting, chlorosis, necrosis and severe defoliation, stem without force. | 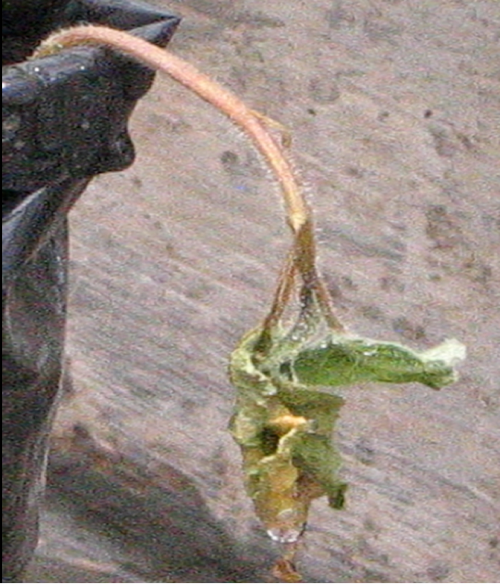 |
|  | 9 | ≥91 -≤ 100 | Dead plant |  |
