# Supplementary material for: Identification of Immunity Related Genes to Study the Physalis peruviana – Fusarium oxysporum Pathosystem
Source: PLoS One. 2013 Jul 3;8(7):e68500. doi: 10.1371/journal.pone.0068500 (PMC3701084; doi:10.1371/journal.pone.0068500)
Supplement: Table S2 — Primers designed for the identification of immunity related genes in Cape gooseberry. (DOCX) [file pone.0068500.s002.docx]

**Table S2:** Primers designed for the identification of immunity related genes in Cape gooseberry.

| **Pritein ID**^§^ | **Domains** | **Isotig** *^ε^* | **Primer ID** | **Primer** | | **Amplicon Size** | | | | | |
| --- | --- | --- | --- | --- | --- | --- | --- | --- | --- | --- | --- |
|  |  |  |  | **Forward** | **Reverse** | **047-4* (P.p)** ^δ^ | **089-1 (P.p)** ^δ^ | **099-1 (P.p)** ^δ^ | **141-1 (P.f)**^Ψ^ | **178-4 (S.a)**^φ^ | **279-4 (P.p)** ^δ^ |
| 224146319 | LRR-Pkinase | JO127269 | PpIRG-1 | CAGCAAATCTTGGTCGGTTT | CCCCCGAGAAGTCATTGTTA | 420 | 420 | 420 | 420 | 420 | 420 |
| 5918254 | NBS-LRR | JO131689 | PpIRG-2 | GCGGAATATGCTAGCTCAGG | AAACACGGTTTTAGGCGATG | 340 | 340 | 340 | NA**^ϕ^** | NA | 340 |
| 225443276 | LRR-Pkinase | JO133959 | PpIRG-3 | ACGTTTGGGAGCTTGAGAGA | GCCGTTTCTGCTCCACTTAG | 530 | NA | 530 | 530 | NA | 530 |
| 225469045 | TIR-NBS | JO140752 | PpIRG-4 ^β^ | TCGCTTGGGTCTAGCCTTTA | CCATAGAGGAGCTGCGTTTC | 480 | 480 | 480 | 480 | 480 | 480 |
| 225463436 | LRR-Pkinase | JO125389 | PpIRG-5 ^β^ | AGCAGGACCAGAGAGCATGT | CACCGGAAATTTCAATGCTT | 500 | 500 | 500 | 500 | 500 | 500 |
| 15418714 | NBS | JO140036 | PpIRG-6 | ATGTCCTGCACGAATCCTTC | CTCACACGAGACGAGAGCTG | 380 | 380 | 380 | 380 | NA | 380 |
| 38489219 | TIR-NBS-LRR | JO132049 | PpIRG-7 | AGTCCAAGGATCGAAGAGCA | ACGACCTCCAACTTCCATTG | 240/400/500 | 240/400/500 | 240/400/500 | 240/500 | 240/310/500 | 240/400/500 |
| 224136153 | LRR | JO140362 | PpIRG-8 | GTCTTGGGAATTGTGCCAGT | AATGGACAACCCGACAAAAA | 330 | 330 | 330 | 330 | 330 | 330 |
| 224136153 | LRR | JO140362 | PpIRG-9 | AATGCTTGCGGGTAAGTGAC | AACTGCCATGTGGATGATGA | NA | 380 | NA | NA | NA | 380 |
| 256542431 | NBS | JO131869 | PpIRG-10 | CGGGAAAACGACTCTTGCTA | CCTCCCTTCCAAACACCTTT | 440 | 440 | 440 | 440 | 440 | NA |
| 37781226 | TIR-NBS-LRR | JO144759 | PpIRG-11 | GAGTGGAGTGGGGAAAACAA | TGACTCTACTTCCCGGACCA | 320 | 320 | 320 | NA | NA | 320 |
| 225423753 | LysM | JO142272 | PpIRG-12 | TCGAAAGGGAGGATCACATT | CAAGCCAATCGGAATAGGAC | 390 | NA | 390 | 390 | NA | 390 |
| 26190254 | CC-NBS-LRR | JO139230 | PpIRG-13 | AAACTGCCCTGCTGAACTGT | ACCCTTCAGCCATCAACAAC | 320 | NA | 320 | NA | NA | NA |
| 26190254 | CC-NBS-LRR | JO139230 | PpIRG-14 | GGAATGCCAGGACTTGGTAA | ACAGTTCAGCAGGGCAGTTT | 450 | NA | NA | NA | 450 | NA |
| 54397639 | LRR | JO142882 | PpIRG-15 | CGTCCCGTTCAGCTTATTGT | TTGGAAAGCTACGGGGTTTA | 400 | 400 | 400 | 400 | NA | NA |
| 255553307 | TIR-NBS-LRR | JO127189 | PpIRG-16 | GTCAAGGCATCCGATTGATT | CATTTTGATGATGCCGACAC | 500 | NA | 500 | 500 | 500 | 500 |
| 34761800 | LRR | JO133125 | PpIRG-17 | TCAATAGGGCCAGAAATTCG | CTGGTCATGTGATTGCCTTG | 480 | 480 | 480 | 480 | 480 | 480 |
| 34761800 | LRR | JO133125 | PpIRG-18 | TGGACCTTTGAAGATGTGGA | CGAATTTCTGGCCCTATTGA | 480 | 480 | NA | 480 | 480 | 480 |
| 39577520 | LRR | JO138765 | PpIRG-19 | AGTTGGAAGGGTGTGAGGTG | CGGGAAGAGAAATTGGATGA | NA | NA | NA | NA | NA | NA |
| 2792188 | LRR | JO138797 | PpIRG-20 | ATCAGAGCCGCTTCCCAACT | CGCTAGAATCGCTGGATCTC | 300 | 300 | 300 | 300 | NA | 300 |
| 255545702 | LRR-Pkinase | JO126441 | PpIRG-21 | GCACAATCCCACCACAAATC | TTGGGAATTTGACCAGAGAGA | 450 | 450 | 450 | 450 | 450 | 450 |
| 255571222 | LRR-Pkinase | JO133019 | PpIRG-22 | AAATTGCCAATGGACTGAGG | GCCTCGTGGATTATGTAGCC | 460 | 460 | 460 | 460 | NA | 460 |
| 66737320 | NBS | JO130165 | PpIRG-23 | GCCAAGGATCTCTTCTGCAC | AAGATGGCCATTGCAGTCA | 490/630 | NA | 490/630 | 630 | NA | 490/630 |
| 66737320 | NBS | JO130165 | PpIRG-24 | TTCTTTCTGCTCCTGGTGGT | GCATCCTTCTCGCTCAAAAG | 800 | 850 | 850 | 800 | NA | NA |
| 255559719 | LRR-Pkinase | JO134183 | PpIRG-25 | CCCTGTAGCTGGTTGTGTGA | GTCCTTGAGCAGTTGCGTTA | 1100/1200 | 1100/1200 | 1100/1200 | 1100/1200 | 1100 | NA |
| 74040324 | NBS | JO129516 | PpIRG-26 | CTTGCCCGCTATTTGCTTAC | GATGTTCGCTTGTGGTGTTG | 400 | 400 | 400 | 400 | NA | 400 |
| 83630761 | CC-NBS | JO138882 | PpIRG-27 | CAGACGAGGAAAACCACCAT | ACATTGCCTCCCACTAAACG | 470 | 470 | 470 | NA | NA | 470 |
| 83630761 | CC-NBS | JO138882 | PpIRG-28 | CGTTTAGTGGGAGGCAATGT | AATCGTACGAGGGCAACAAC | 470 | NA | 470 | 470 | NA | NA |
| 255558508 | Pkinase | JO132753 | PpIRG-29 | CTTGTTCATGGACGAGACGA | TTGGAAGCTGAAGTGCCTTT | 490 | 490 | 490 | 490 | NA | 490 |
| 8547237 | CC-NBS | JO133481 | PpIRG-30 ^β^ | AATCTGGAGGCTGCATTGTAA | CATTTGAAGGTGATGCATGG | 310 | 310 | 310 | 310 | 310 | 310 |
| 142942427 | CC-NBS | JO124419 | PpIRG-31 ^β^ | CAAGCTGCGCCATCTACATA | AGGCATGGAAAAGCATCATC | 510 | 510 | 510 | 510 | 510 | 510 |
| 4689223 | CC-NBS-LRR | JO142447 | PpIRG-32 ^β^ | CTGATCCGGAGAGTGGTTTC | ACGCTCCAAATCAAAGGTTG | 490 | 490 | 490 | 490 | 490 | 490 |
| 255561034 | CC-NBS-LRR | JO133251 | PpIRG-33 ^β^ | TGGATTCAAGCCTGCAAAAT | CCTCCAACATTAAGCCCTCA | 410 | 410 | 410 | 410 | 410 | 410 |
| 164598916 | NBS-LRR | JO134175 | PpIRG-34 | ATCTCGGCTCCACCTCATAG | TTGGGAAGGCATGAAAAGAC | 440 | 440 | 440 | 440 | NA | 440 |
| 224111328 | TIR | JO136499 | PpIRG-35 | CCTTTGCCTCATTCCCATTA | GGGCTAACGACCTTCAAAGA | NA | NA | NA | NA | NA | NA |
| 126843151 | LRR-Pkinase | JO138575 | PpIRG-36 | TTGCAGGCTTTGGAGAACTT | TGGTTAAATGTTGGCTGAAGG | 500 | 500 | 500 | NA | NA | 500 |
| 126843151 | LRR-Pkinase | JO138575 | PpIRG-37 | GGAAGAAGAAGGAGGCTGCT | TCCAATGGCAATTTTCCTTC | 500 | 500 | 500 | 500 | NA | NA |
| 224111082 | LysM | JO141781 | PpIRG-38 | AGAGGGCCAGAGTTTGTTGA | CCAAATCTTGAAGCCATTGAA | 470 | 470 | 470 | 470 | NA | 470 |
| 225429554 | Pkinase | JO140379 | PpIRG-39 | ACTGCATCGTCCACCTATCC | TATGATCTGCCCAAAGGAGG | 1100 | 1100 | 1100 | 1100 | 1100 | NA |
| 255560213 | Pkinase | JO130045 | PpIRG-40 | GGGGAACTTTGGCAACTGTA | GACCAGCACTGTTCCATCAA | NA | NA | NA | 540/600/650 | NA | NA |
| 75261541 | NBS | JO140158 | PpIRG-41 | GTAAGGGAAGGACGAATGCA | CGAAGCTTATGGGGATCACT | 500 | 500 | 500 | NA | NA | 500 |
| 6456755 | NBS-LRR | JO126009 | PpIRG-42 | GCTCCCTGCATTGTTTTCA | TGCCTGGAAAGAGTCAACAA | 510 | 510/540 | 510/540 | 510/540 | 510/540 | 510 |
| 193795975 | NBS | JO125561 | PpIRG-43 ^β^ | AACATGGGATGAAACACTTGC | TGAAAAGCGTGCCAACTAAA | 320 | 320 | 320 | 320 | 320 | 320 |
| 2258317 | CC-NBS-LRR | JO131403 | PpIRG-44 ^β^ | TGATGAGTGGGATGACCTGA | GCTCAACATCAACGCTGGTA | 390 | 390 | 390 | 390 | 390 | 390 |
| 37781356 | TIR-NBS-LRR | JO138542 | PpIRG-45 | GCGAGTTGAAGAGGTTGGAG | CATTCGGGAGATAGCTTGGA | 650 | 650 | 650 | 650 | 650 | 650 |
| 37781356 | TIR-NBS-LRR | JO138542 | PpIRG-46 ^β^ | TTCTGCTGCCACTGATGTTC | GAGACCCCAAGCACATTTCA | 520 | 520 | 520 | 520 | 520 | 520 |
| 225463524 | NBS | JO145025 | PpIRG-47 | TTTGGTCTATGGGTGGTTGG | TCTTCTTCGCTCTTCCATGC | NA | NA | NA | NA | NA | NA |
| 225458497 | NBS-LRR | JO139035 | PpIRG-48 | CGCCACCGAACTCATAAAAT | CTAAACACCCTCCTCCACCA | 1300 | 1300 | NA | NA | NA | 1300 |
| 225458497 | NBS-LRR | JO139035 | PpIRG-49 | ACGAGGTTGCCAAAAGAATG | GAGAGCTGTGGGAACAAACC | 500 | 500 | 500 | 500 | NA | 500 |
| 255542420 | TIR-NBS-LRR | JO125219 | PpIRG-50 | AACGCCGGTATTCGAACTTT | GGCTAAGCACATTCCTTCCA | 390 | 390 | 390 | NA | 390 | 390 |
| 109289912 | NBS-LRR | JO142950 | PpIRG-51 ^β^ | GCGTTTGGGCTAGATAGGAA | GGCCAGGTCATGAACAAGAT | 480 | 480 | 480 | 480 | 480 | 480 |
| 224115778 | NBS-LRR | JO138810 | PpIRG-52 | ATTTTCAATTGACCCCGAAG | CGGTTGCTGAGATGAATGAA | 480 | NA | 480 | 480 | 480 | NA |
| 224115778 | NBS-LRR | JO138810 | PpIRG-53 ^β^ | TCCATCAGGCAAAGCAGATA | GCAAGTGCCCCAGTCTAATC | 520 | 520 | 520 | 520 | 520 | 520 |
| 224111284 | CC-NBS | JO139489 | PpIRG-54 | TATCGACCACTGGAGCTGAG | CTGCGGCTTGTCACATTCTA | 460 | NA | 460 | 460 | 460 | 460 |
| 224130338 | CC-NBS-LRR | JO142897 | PpIRG-55 | CCCATCTTGAAGCGTTGTTT | CGAGAGCAAGTGTTCAGCAA | 430 | 430 | 430 | 430 | NA | NA |
| 224130338 | CC-NBS-LRR | JO142897 | PpIRG-56 ^β^ | CATGCATAGGTTGGGACAAA | AGCCTCCAACTCGTGAAAGA | 490 | 490 | 490 | 490 | 490 | 490 |
| 255567754 | TIR-NBS | JO144821 | PpIRG-57 | ATTTTCCAAATGCCATCCTG | GGGGGATCACAAGAGTCTCA | NA | NA | NA | NA | NA | NA |
| 224117254 | NBS-LRR | JO130639 | PpIRG-58 | CGAGAAGGTGTGAGATGCAA | AGACTTGGCCAGCTTGAAAC | 530 | 530 | 530 | NA | NA | 530 |
| 255578636 | CC-NBS-LRR | JO130641 | PpIRG-59 | TCAAATGCCCAATCTTCCTC | CCACTTGGGCTCATCATTTT | 480 | 480 | 480 | 480 | 480 | 480 |
| 255578636 | CC-NBS-LRR | JO130641 | PpIRG-60 ^β^ | TGAACAACTCCCAAGCATCA | GAGGAGATGGCAAAACGAAA | 490 | 490 | 490 | 490 | 490 | 490 |
| 224122448 | TIR-CC-NBS-LRR | JO133073 | PpIRG-61 ^β^ | GAAGTTCGCATGGTTGGTCT | TGTGGCGGAGAAAGACTGTT | 490 | 490 | 490 | 490 | 490 | 490 |
| 224122448 | TIR-CC-NBS-LRR | JO133073 | PpIRG-62 | ACGAGCAAAAACTGGTACCG | TAAAGAATCGCACCCGCTAA | 550 | 550 | 550 | NA | 550 | 550 |
| 225449965 | CC-NBS-LRR | JO134053 | PpIRG-63 ^β^ | CCTTGTTACCTGGTGGCATT | GCCAAATCCTTTTCACCAGA | 500 | 500 | 500 | 500 | 500 | 500 |
| 225469040 | NBS-LRR | JO134974 | PpIRG-64 | GATTCCATCATCGAGGCAAT | TGATGTTGTTGGAAGGAGGA | NA | 490 | NA | 490 | 470/490 | 490/600/650 |
| 225469040 | NBS-LRR | JO134974 | PpIRG-65 | TCCTCCTTCCAACAACATCA | TTGAAAGACATGGCAGCAGA | NA | 600/800 | NA | 500 | 500 | NA |
| 225462769 | NB-LRR | JO139181 | PpIRG-66 | AACAATCTCTCCGCATCACC | TGACCACCGCACCTGTATTA | 500 | 500 | 500 | 500 | 500 | 500 |
| 225469640 | NBS-LRR | JO139995 | PpIRG-67 | GCGGTTTCAGTGTTCGGTAT | TGATCGAAAAGCCTTCTTGC | NA | 460 | 460 | 460 | 460 | 460 |
| 148923085 | LRR-Pkinase | JO134491 | PpIRG-68 | GGAACAGGACCTACGAGACG | CACAGTCTGCGAACCAACTT | NA | NA | NA | NA | NA | NA |
| 225429912 | LRR-Pkinase | JO138791 | PpIRG-69 | GAAAATGGCCAGCATAGGAT | GTGGGATTCTTGAGCCTTGA | 410 | 410 | NA | 410 | 410 | 410 |
| 558887 | TIR-NBS-LRR | JO129083 | PpIRG-70 | TCAAATGGGTCAAGATGTGG | TTTTAATCGACGGCTCATCC | NA | NA | NA | NA | NA | NA |
| 225430436 | LRR | JO128555 | PpIRG-71 | TAGATAACGCGAGGGAGGTT | AGCAACAAGATCCGAGGACA | 440 | NA | 440 | 440 | 440 | 440 |
| 225430436 | LRR | JO128555 | PpIRG-72 | TGGTTGTGTGACAGGTTCAA | CATTTTCAAGGCACCATTCC | 410 | 410 | 410 | 410 | 410 | 410 |
| 255556695 | LRR-Pkinase | JO134019 | PpIRG-73 | GGGGATAGTTGGGAAGGAGT | CAAGCATATCAAGGCCAGGT | 1200 | NA | 1200 | NA | 1200 | 1200 |
| 224141079 | LRR-Pkinase | JO138596 | PpIRG-74 | CCAACTGCCAGGAAAATCTC | CGGGCAATTTCTTGAGTGAT | 400 | 400 | 400 | 400 | 400 | 400 |
| 224141079 | LRR-Pkinase | JO138596 | PpIRG-75 | AGCTGCGATGGTATTTGTCC | TTTGCCATCGGAGTTTAAGG | 440 | 440 | NA | 440 | 440 | 440 |
| 255551969 | LysM-Pkinase | JO143107 | PpIRG-76 | TCTTGCTGTGACTTGCCTTG | GGGTGCAATGCCATAACAAT | 430 | NA | NA | NA | 430 | NA |
| 224115870 | LysM-Pkinase | JO133387 | PpIRG-77 | AGGGTTCAAATTGCTCTGGA | GATTCTCATGGGTGCAAGCT | NA | NA | NA | NA | NA | NA |
| 255557731 | LRR-Pkinase | JO132591 | PpIRG-78 | CACCTGCACAAACATCCATT | CCTGATGAGTTGGGAAACCT | NA | NA | NA | NA | NA | NA |
| 225424831 | TIR-NBS-LRR | JO127789 | PpIRG-79 ^β^ | GGTTGCATGGAATAGGAGGA | TTCAGAAAAGTTGGGGATGG | 500 | 500 | 500 | 500 | 500 | 500 |
| 225461003 | CC-NBS-LRR | JO134149 | PpIRG-80 | CGGAGAGCCAAGAAGACAAC | CCTCAAGATCAAGCCTGGAC | 490 | 490 | NA | NA | 490 | 490 |
| 225439697 | LRR-Pkinase | JO138689 | PpIRG-81 | CCGGTGAGGTTGTTATGTGA | GGACATCGGCAACAATTTCT | NA | NA | 600 | NA | 650 | 650 |
| 225439697 | LRR-Pkinase | JO138689 | PpIRG-82 | AGAAATTGTTGCCGATGTCC | TTTATTGGAGGGCGAGATTC | 420 | 420 | NA | NA | 420 | 420 |
| 225451885 | LRR-Pkinase | JO139507 | PpIRG-83 | CCTTCAAGCATTCGGACAAT | AGGGATGTAAAGGCAGCAAA | 750 | 750 | NA | 750 | 750 | 750 |
| 225451885 | LRR-Pkinase | JO139507 | PpIRG-84 | GCCAAATACAAGCGCAATTT | TCCTAGCATTGGCAATTTGA | 450 | 450 | NA | NA | 1000 | 450 |
| 225430870 | LysM-Pkinase- | JO134217 | PpIRG-85 | GGACTTTTGGCAAACTCTGG | GTGGCTGCTTTGACCTCTTC | NA | NA | NA | 400 | 400 | NA |
| 225430870 | LysM-Pkinase | JO134217 | PpIRG-86 | GTTTTCCTCCTCCCACTTCC | GCTTCAACATGGCTTTGTCC | 400/450 | NA | 400/450 | NA | 400/450 | 400/450 |
| 256260670 | NBS | JO140832 | PpIRG-87 | CATCGACTCTTTCAGCCACA | TTGTTGGGTTTCAGGAGGTC | NA | 460 | 460 | 460 | 460 | 460 |
| 256260670 | NBS | JO140832 | PpIRG-88 | GACCTCCTGAAACCCAACAA | CAAAGATGCCCAAAGAAAGC | 400 | 400 | NA | 400 | 400 | 400 |
| 56406364 | CC-NBS | JO138325 | PpIRG-89 | GTTGAGCGCGTCTATGGTTT | CCCCTGACAAATATGGATGG | 430 | 430 | NA | 430 | 430 | 430 |
| 256260664 | NBS | JO129491 | PpIRG-90 ^β^ | GGCCATCCACAACCGTATTA | AGGAGGATGTCGCTGAAAGA | 430 | 430 | 450 | 430 | 430 | 430 |
| 255569098 | CC-NBS | JO133195 | PpIRG-91 | TCACCGAGCTGCATAATCTG | CAGCACGAGATGTTCCAAGA | 500 | 500 | 500 | 500 | 500 | 500 |
| 224081190 | LRR-Pkinase | JO138807 | PpIRG-92 | GGCCTCACTCTTCCAAACCT | GCTGCCGTTGAGGTAGTTTC | 450 | NA | 450 | 450 | NA | 450 |
| 224081190 | LRR-Pkinase | JO138807 | PpIRG-93 | ATTGGCTGCATCCTGAAAAC | GTTTTCGTCTTCCTCGTCCA | 600 | 600 | 600 | 600 | NA | 600 |
| 255561552 | NBS | JO140517 | PpIRG-94 | TTGATCCTTCCAGTCCGTTC | TGAATGAATGTGGAGGCTTG | NA | NA | NA | NA | NA | NA |

^§^ GenBank protein ID; *^ε^* GenBank Cape gooseberry ID; ^δ^ *Physalis peruviana*; ^Ψ^ *Physalis floridana*; ^φ^ *Solanum auriculatum*; ^β^ Markers selected for preliminary association studies; **^ϕ^** No Amplification, * Only the 4 last identifiers for each genotype are specified (compare with Table 1).
