# Supplementary material for: Identification of Immunity Related Genes to Study the Physalis peruviana – Fusarium oxysporum Pathosystem
Source: PLoS One. 2013 Jul 3;8(7):e68500. doi: 10.1371/journal.pone.0068500 (PMC3701084; doi:10.1371/journal.pone.0068500)
Supplement: Table S3 — Number of reads per marker sequenced by 454 on the 14 Physalis genotypes. (DOCX) [file pone.0068500.s003.docx]

**Table S3:** Number of reads per marker sequenced by 454 on the 14 *Physalis* genotypes.

| **Marker/**  **Accession** | **09U047-1** | **09U047-4** | **09U063-7** | **09U071-4** | **09U086-4** | **09U089-1** | **09U099-1** | **09U139-1** | **09U141-1** | **09U173-3** | **09U210-6** | **09U216-6** | **09U274-3** | **09U279-4** |
| --- | --- | --- | --- | --- | --- | --- | --- | --- | --- | --- | --- | --- | --- | --- |
| **PpIRG-4** | 64 | 55 | 28 | 65 | 154 | 17 | 96 | 27 | 47 | 78 | 37 | 44 | 32 | 211 |
| **PpIRG-5** | 15 | 2 | 9 | 507 | 0 | 38 | 291 | 0 | 23 | 31 | 27 | 46 | 24 | 26 |
| **PpIRG-30** | 78 | 71 | 78 | 59 | 82 | 92 | 302 | 42 | 122 | 63 | 186 | 72 | 41 | 0 |
| **PpIRG-31** | 144 | 31 | 200 | 25 | 45 | 27 | 68 | 17 | 32 | 129 | 36 | 46 | 20 | 39 |
| **PpIRG-32** | 29 | 17 | 64 | 32 | 52 | 10 | 129 | 30 | 31 | 59 | 48 | 19 | 70 | 342 |
| **PpIRG-33** | 21 | 25 | 65 | 115 | 39 | 47 | 72 | 84 | 85 | 240 | 70 | 94 | 31 | 29 |
| **PpIRG-43** | 59 | 91 | 174 | 156 | 166 | 123 | 84 | 98 | 200 | 92 | 92 | 182 | 96 | 100 |
| **PpIRG-44** | 111 | 53 | 69 | 57 | 87 | 43 | 119 | 94 | 90 | 369 | 88 | 110 | 58 | 83 |
| **PpIRG-46** | 47 | 148 | 43 | 42 | 61 | 47 | 82 | 23 | 6 | 67 | 17 | 34 | 53 | 88 |
| **PpIRG-51** | 89 | 17 | 106 | 42 | 104 | 48 | 115 | 96 | 43 | 24 | 46 | 71 | 18 | 43 |
| **PpIRG-53** | 38 | 44 | 23 | 21 | 86 | 8 | 126 | 44 | 35 | 52 | 45 | 41 | 12 | 32 |
| **PpIRG-56** | 42 | 2 | 37 | 186 | 54 | 15 | 171 | 42 | 66 | 66 | 72 | 93 | 28 | 159 |
| **PpIRG-60** | 52 | 29 | 88 | 31 | 113 | 26 | 113 | 58 | 67 | 78 | 83 | 115 | 30 | 171 |
| **PpIRG-61** | 37 | 43 | 146 | 16 | 87 | 41 | 117 | 186 | 54 | 342 | 114 | 22 | 28 | 18 |
| **PpIRG-63** | 220 | 27 | 183 | 36 | 66 | 50 | 401 | 23 | 29 | 46 | 250 | 15 | 12 | 2 |
| **PpIRG-79** | 29 | 2 | 82 | 19 | 31 | 33 | 97 | 57 | 179 | 42 | 28 | 15 | 30 | 31 |
| **PpIRG-90** | 111 | 80 | 102 | 58 | 108 | 75 | 200 | 109 | 48 | 166 | 51 | 69 | 74 | 79 |
